# Cancer care barriers and interventions among immigrant workers: an evidence map and practice-oriented review

**DOI:** 10.3389/fonc.2026.1922610

**Published:** 2026-09-14

**Authors:** Youssef Samaha, Roshan Asrani, Erin Jay Feliciano, Edward Christopher Dee, Oikigbeme Oikeh, Theodora Anagnostou

**Affiliations:** 1 Department of Medicine, Icahn School of Medicine at Mount Sinai; NYC Health + Hospitals/Elmhurst, Queens, NY, United States; 2 Department of Hematology/Oncology, Icahn School of Medicine at Mount Sinai, New York, NY, United States; 3 Department of Radiation Oncology, Memorial Sloan Kettering Cancer Center, New York, NY, United States

**Keywords:** cancer care delivery, cancer screening, health equity, immigrant workers, limited english proficiency, oncology disparities, patient navigation, public charge

## Abstract

**Background:**

Immigrant workers represent a distinct oncology equity population because cancer care barriers occur at the intersection of migration status, occupational precarity, language access, fragmented insurance eligibility, legal vulnerability, income loss, and limited paid leave. Oncology literature rarely characterizes immigrant workers as a distinct analytic group.

**Objective:**

To map direct, partial, and indirect evidence on cancer care barriers and interventions relevant to immigrant workers and translate the findings into evidence-supported implications, evidence-informed adaptations, and research or policy directions.

**Methods:**

We conducted a modified, practice-oriented evidence map using 48 sources identified through targeted database, citation, and policy-source searching. Sources were charted by population, immigrant-worker specificity, source type, primary analytic domain, cancer continuum stage, barrier or intervention focus, key contribution, and evidence gap.

**Results:**

Of 48 mapped sources, 1 was direct, 8 were partial, and 39 were indirect evidence. The source set comprised 20 empirical studies, 17 evidence syntheses, 9 government, policy, or legal sources, and 2 commentaries or methods papers. Evidence was concentrated in structural and policy access, screening and navigation, and language and communication; evidence remained sparse for work-related treatment interruption, survivorship and return to work, employer interventions, and clinical trial access. Professional interpretation, language-concordant communication, patient navigation, community health worker outreach, culturally tailored screening, and community-based participatory approaches had the clearest support in adjacent or broader immigrant populations. Flexible scheduling, bundled visits, workplace protections, and structured return-to-work support were research or policy priorities requiring evaluation.

**Conclusions:**

Immigrant workers are a structurally vulnerable oncology population shaped by overlapping employment, legal, linguistic, financial, and health-system barriers. Oncology systems can implement supported language-access and navigation strategies while testing worker-adapted delivery models and advocating for policy changes that reduce coverage and employment-related barriers.

## 1 Introduction

Foreign-born workers accounted for 19.1% of the U.S. civilian labor force in 2025 and were more likely than native-born workers to be employed in service occupations; natural resources, construction, and maintenance occupations; and production, transportation, and material-moving occupations (53). Many immigrant workers also work in agriculture, food services, domestic work, caregiving, cleaning, and other low-wage or informal occupations (1, 2). Despite this substantial economic participation, many face persistent barriers to healthcare access across cancer prevention, screening, diagnosis, treatment, survivorship, and palliative care (1–4). These barriers are produced by the intersection of legal vulnerability, language discordance, fragmented insurance eligibility, income instability, transportation barriers, fear of job loss, lack of paid medical leave, and limited institutional navigation support (1–4).

For this review, immigrant workers were operationally defined as foreign-born persons engaged in paid work, including formal, informal, hourly, seasonal, migrant, or low-wage employment, regardless of documentation status. Because oncology studies rarely measured all of these attributes simultaneously, adjacent immigrant-relevant populations were included using the direct, partial, and indirect evidence tiers described below. The term oncology equity population refers here to a group at risk for systematic and avoidable differences in cancer access, care delivery, or outcomes because of structural conditions; it is an analytic framing rather than a legal or benefit-eligibility category.

Immigrant workers should be considered separately from broad immigrant categories that may obscure the occupational and economic conditions shaping cancer care. A salaried, insured immigrant professional and an undocumented hourly worker may both be categorized as immigrants, but their ability to attend screening, complete diagnostic evaluation, start treatment, tolerate toxicity, and maintain follow-up may differ substantially. For workers in low-wage or informal sectors, attending oncology visits may require sacrificing wages, risking termination, losing childcare coverage, or navigating immigration-related fears within mixed-status households (1–4).

The Affordable Care Act expanded coverage for many U.S. residents but left major exclusions for undocumented immigrants, including exclusion from federally subsidized marketplace plans and most federally funded Medicaid coverage except emergency services and selected state-funded programs (3, 4). Many newly arrived lawful permanent residents also remain subject to a five-year Medicaid waiting period in most states. Uninsured and underinsured immigrant workers therefore often rely on safety-net systems, emergency Medicaid, federally qualified health centers, public hospitals, and charity care programs. These systems are essential but may be fragmented and poorly aligned with longitudinal, multidisciplinary oncology treatment (1–4, 47).

Cancer care is uniquely vulnerable to these access barriers because it requires timely movement through a complex care system. Screening must be followed by diagnostic confirmation; diagnosis by staging, specialty referral, treatment planning, financial clearance, and treatment initiation; and treatment by repeated visits, laboratory testing, imaging, supportive care, and toxicity management. Survivorship requires surveillance, rehabilitation, psychosocial support, and return-to-work planning, whereas palliative and end-of-life care require accurate communication, trust, and culturally responsive family-centered decision-making. Barriers at any point can produce delays, incomplete treatment, avoidable suffering, and inequitable outcomes (7–10, 46).

Prior literature documented disparities among immigrants, undocumented patients, foreign-born populations, migrant people, and patients with limited English proficiency (1, 2, 44). A meta-analysis found that migrants were less likely than non-migrants to be diagnosed with early-stage cancer overall (odds ratio 0.84, 95% confidence interval 0.78-0.91), with statistically significant differences for breast and prostate cancer (7). Studies of patients with limited English proficiency identified disparities in communication, treatment understanding, oncology outcomes, and, in end-of-life settings, care that may not align with patients’ values (9, 10, 33, 36). Studies of undocumented immigrants and safety-net populations highlighted insurance exclusion, delayed access, fragmented pathways, and variable state-level mechanisms (2, 43). However, relatively few oncology studies isolated immigrant workers as a distinct analytic group.

This review therefore used a modified evidence-map approach to characterize the evidence base, distinguish worker-specific from adjacent evidence, map barriers across the cancer care continuum, and link findings to multilevel oncology actions. **Figure 1** presents the worker-centered conceptual framework used to organize barriers and action levels. The central objective was to use adjacent evidence without overstating its specificity while identifying the measurements and interventions required for worker-specific oncology research.

**Figure 1:**
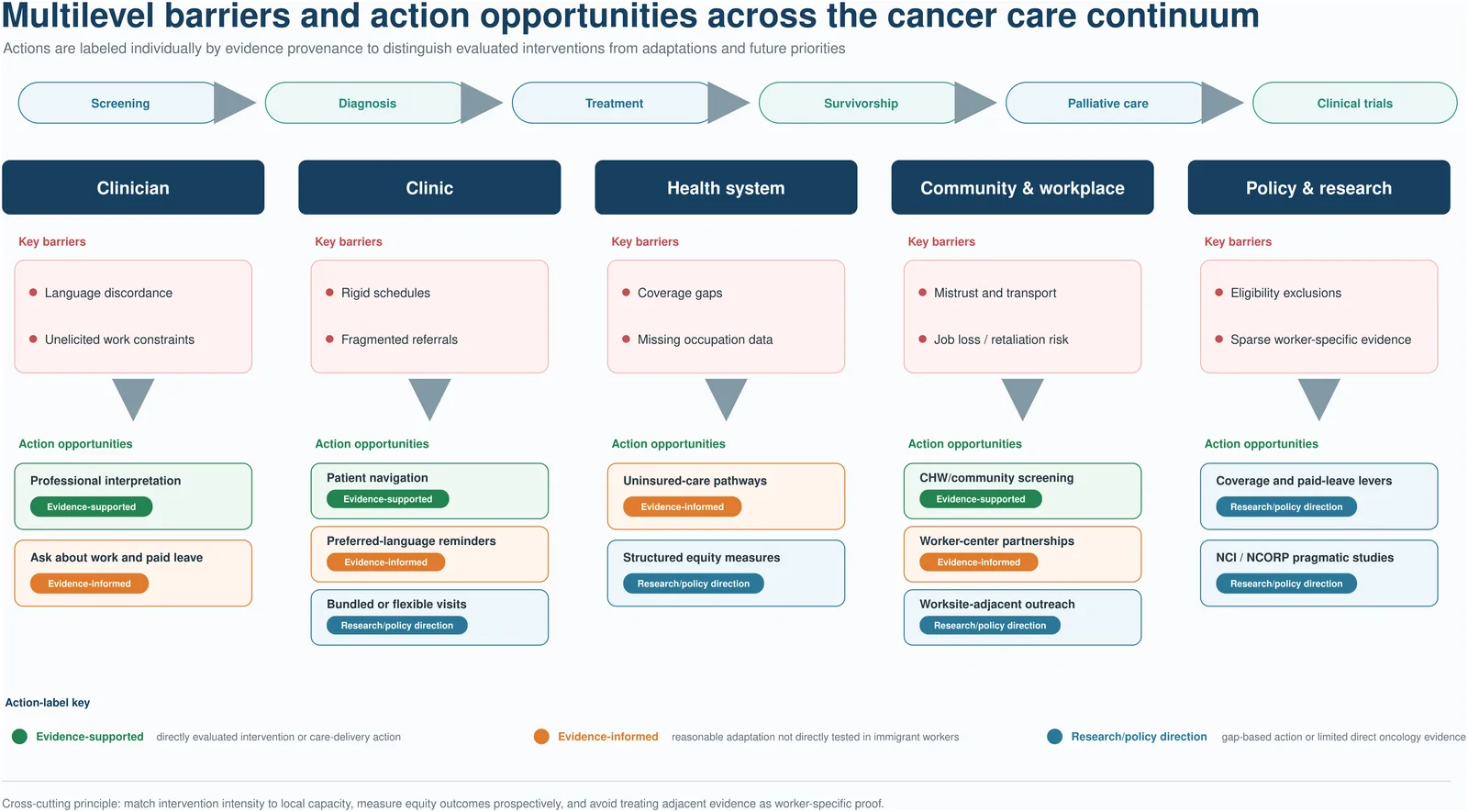
Multilevel conceptual framework for barriers and action opportunities across the cancer care continuum. The framework shows how structural and coverage, language and communication, workplace and legal, and psychosocial barriers can interrupt screening, diagnosis, treatment initiation and continuity, survivorship, palliative care, and clinical trial access. Actions are labeled individually as evidence-supported, evidence-informed, or research/policy directions according to the Methods definitions. The framework is a conceptual synthesis and not a quantitative causal model.

## 2 Methods: evidence-map approach

### 2.1 Design and rationale

Evidence maps systematically organize broad, heterogeneous bodies of literature across predefined dimensions to show concentrations of evidence and gaps, commonly through matrices, tables, or visual frameworks (48, 49). We used a modified, practice-oriented evidence map because the literature spanned multiple populations, study designs, cancer types, policy contexts, and care-continuum domains, while few studies directly identified immigrant workers. The modifier practice-oriented does not denote a novel evidence-synthesis methodology; it describes the added translation step linking mapped findings to oncology care-delivery implications.

This approach differed from a narrative review by using explicit eligibility criteria, a documented search process, structured source charting, predefined specificity tiers, and quantified domain counts. It also differed from a formal scoping review because it did not use a registered protocol, exhaustive multi-database searching, duplicate independent screening, or PRISMA-ScR reporting. A rapid review was not selected because rapid reviews typically streamline a conventional systematic review to answer a focused, time-sensitive question, often concerning intervention effectiveness, whereas our objective was broad descriptive mapping across multiple populations, barriers, interventions, and policy contexts (50).

### 2.2 Source identification and selection

YS conducted targeted searches of PubMed and Google Scholar during June 2026, with no publication-date restriction and a literature cutoff of June 28, 2026. Official policy and guidance sources were identified from the U.S. Department of Health and Human Services, Department of Homeland Security and Federal Register, Centers for Medicare & Medicaid Services, Kaiser Family Foundation, and Immigrant Legal Resource Center websites. Reference lists of key reviews and included sources were screened for additional eligible publications. A targeted policy update was repeated on August 4, 2026 and added the July 2026 Department of Homeland Security final rule on public charge (51).

Search concepts combined population terms (immigrant, migrant, foreign-born, undocumented, limited English proficiency) with worker terms (worker, farmworker, seasonal, occupation, employment, paid leave, workplace accommodation, return to work) and oncology terms (cancer, oncology, screening, diagnosis, treatment, survivorship, palliative care, clinical trial). Examples included: (immigrant OR migrant OR foreign-born OR undocumented) AND (worker OR farmworker OR seasonal OR occupation OR employment) AND (cancer OR oncology); (immigrant OR undocumented OR foreign-born) AND (cancer screening OR diagnosis OR treatment OR survivorship OR palliative care); (limited English proficiency OR language access OR interpreter) AND (cancer OR oncology); and public charge AND (healthcare OR Medicaid OR cancer).

The source set was developed iteratively from targeted topic searches, citation chaining, and known high-relevance policy sources. YS screened source titles and summaries, reviewed full texts when needed, performed initial charting and categorization, and discussed source selection and interpretation with the coauthors. Screening and extraction were not independently duplicated. Sources were included if they addressed cancer prevention, screening, diagnosis, treatment, survivorship, palliative care, clinical trial access, or oncology care delivery in immigrant workers or an adjacent immigrant-relevant population, or if they addressed a policy or intervention directly relevant to cancer access. Sources were excluded if they lacked cancer-care or oncology-access relevance, lacked immigration-related relevance, focused exclusively on race or ethnicity without migration relevance, or addressed occupational carcinogen exposure without linkage to cancer care access or delivery.

### 2.3 Evidence charting and categorization

For each source, we charted the full reference, population and setting, source type, immigrant-worker specificity, primary analytic domain, cancer-continuum stage, barrier or intervention focus, key contribution, relevance to immigrant workers, and evidence gap. We then applied a two-condition operational rule. Direct evidence required a source population explicitly defined jointly by immigrant status and worker or occupational status, such as migrant workers, farmworkers, seasonal workers, or another occupationally defined immigrant group. Partial evidence required (1) a population explicitly defined as immigrant, migrant, undocumented, or foreign-born and (2) measurement, analysis, or substantive discussion of at least one work-related construct—employment, occupation, workplace, work schedule, paid leave, job security, ability to earn, or return to work—or explicit distinction of a worker-defined subgroup. Reporting employment only as a background demographic did not qualify. Indirect evidence informed immigrant cancer care but did not meet both conditions; this included immigrant-defined sources without a substantive work-related construct and broader limited-English-proficiency, uninsured, or policy sources without an explicitly immigrant-defined population. These tiers described population relevance, not methodological quality or certainty.

Each source was assigned to one primary analytic domain for descriptive counting: structural and policy access; language and communication; screening and navigation; diagnosis and treatment continuity; psychosocial care, survivorship, and palliative care; clinical trial access; or data and measurement. Sources could inform additional domains narratively. Because the source types were heterogeneous, we did not apply one pooled risk-of-bias instrument or formal certainty grading. Counts therefore describe the distribution of the mapped literature rather than comparative evidence strength.

### 2.4 Synthesis and evidence-to-action translation

Findings were synthesized across the cancer care continuum and mapped by level of action: clinician, clinic, health system, community, employer or workplace, and policy. Recommendations were classified by provenance using the editor-specified rule. Evidence-supported actions were limited to interventions or care-delivery practices directly evaluated in the mapped evidence. Evidence-informed actions were reasonable adaptations from related evidence that had not been directly tested in immigrant workers. Research or policy directions were based mainly on mapped gaps or areas with limited direct oncology evidence. These labels were applied to individual actions rather than entire domains and did not constitute formal guideline grading.

**Figure 2** depicts the evidence-map workflow from source identification through classification, domain mapping, and evidence-to-action translation. **Figure 1** shows the multilevel conceptual framework. The framework was iteratively refined during charting and organized how structural, language, workplace and legal, and psychosocial barriers could interrupt the care continuum and how action could occur at clinician, clinic, health-system, community, workplace, and policy levels. **Table 1** summarizes the quantified evidence profile and evidence-to-action synthesis, and **Supplementary Table 1** provides the full source-level matrix. This manuscript is presented as a review article using a modified evidence-map approach and is not reported as a formal PRISMA-ScR scoping review.

**Figure 2:**
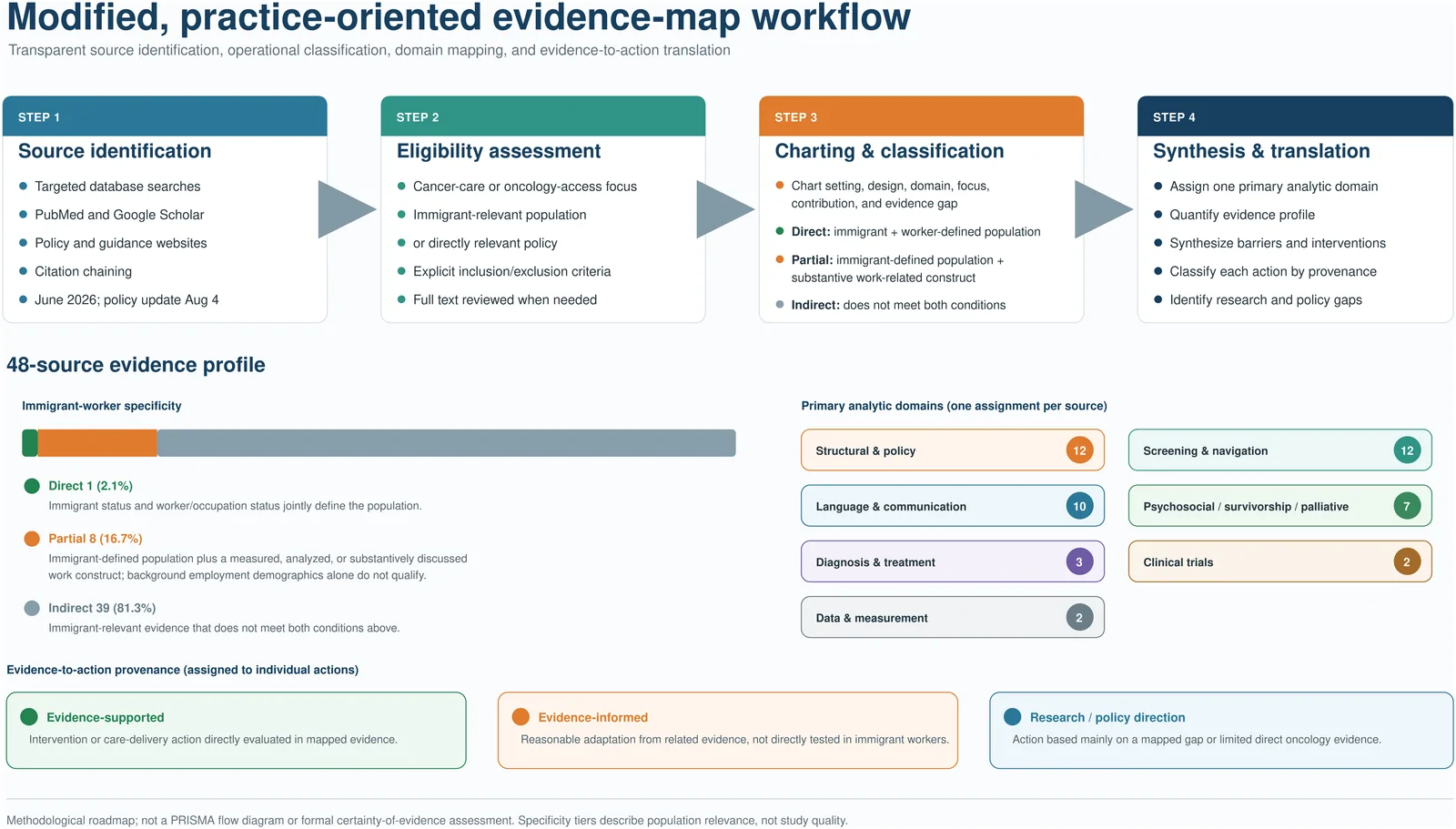
Workflow for the modified, practice-oriented evidence map. The workflow summarizes targeted source identification, eligibility assessment, source charting, operational classification, assignment to primary analytic domains, and evidence-to-action translation. Direct evidence required an occupationally defined immigrant-worker population; partial evidence required both an immigrant-defined population and a substantive work-related construct; indirect evidence did not meet both conditions. The figure is a methodological roadmap rather than a PRISMA flow diagram and does not imply exhaustive screening.

**Table 1:** Evidence profile and evidence-to-action synthesis for immigrant workers across the cancer care continuum.

Domain and evidence profile	Main mapped finding	Action classification and implication	Research or policy direction/gap
Structural and policy access (n=12: 0 direct, 3 partial, 9 indirect)	Coverage exclusion, safety-net fragmentation, cost, transportation, and public-charge uncertainty were consistently represented; workplace intervention evidence was thin.	Evidence-informed: Early coverage and financial counseling, navigation, and clear multilingual policy information are reasonable adaptations from access evidence but were not directly tested in immigrant workers.	Research/policy direction: Evaluate uninsured oncology pathways, paid leave, anti-retaliation protections, and effects on diagnostic and treatment timelines.
Language and communication (n=10: 0 direct, 0 partial, 10 indirect)	Language barriers affected consent, treatment teaching, symptom reporting, and goals-of-care communication.	Evidence-supported in adjacent care-delivery evidence: professional interpretation reduced consequential errors in LEP populations. Evidence-informed: integrate interpretation and language-concordant communication into immigrant-worker oncology workflows.	Research direction: Test how language services affect work-schedule conflicts, missed visits, adherence, and toxicity reporting.
Screening and navigation (n=12: 1 direct, 3 partial, 8 indirect)	Screening disparities were common; navigation, community health workers, culturally tailored outreach, and participatory approaches improved screening.	Evidence-supported in immigrant screening populations: navigation, community health worker outreach, culturally tailored education, and community partnerships improved screening. Evidence-informed: adapt delivery to worker settings.	Research direction: Evaluate worksite-adjacent models, employer or worker-center partnerships, diagnostic completion, and downstream outcomes.
Diagnosis and treatment continuity (n=3: 0 direct, 0 partial, 3 indirect)	Migrants were less likely to receive early-stage diagnosis; direct worker-specific treatment-continuity measures were absent.	Evidence-informed: Navigation and rapid follow-up after abnormal findings are reasonable adaptations from broader oncology evidence but were not directly tested in immigrant workers.	Research direction: Test bundled visits, flexible scheduling, predictable calendars, and missed-visit outreach using treatment-completion outcomes.
Psychosocial, survivorship, and palliative care (n=7: 0 direct, 2 partial, 5 indirect)	Psychosocial distress and end-of-life communication barriers were described; return-to-work outcomes were rarely measured.	Evidence-supported in adjacent immigrant or LEP cancer evidence: psychosocial interventions and interpreter-supported palliative communication were evaluated. Evidence-informed: adapt delivery to worker needs.	Research direction: Develop and evaluate rehabilitation, workplace accommodation, job-retention, and return-to-work pathways.
Clinical trial access (n=2: 0 direct, 0 partial, 2 indirect)	English-language requirements and underrepresentation of patients with limited English proficiency were documented.	Evidence-informed: Reduce avoidable language exclusions and provide translated materials and interpreter-supported consent; these responses to documented barriers were not directly tested in immigrant workers.	Research direction: Measure occupation and work constraints; test transport support, flexible visits, and decentralized procedures.
Data and measurement (n=2: 0 direct, 0 partial, 2 indirect)	Cancer datasets incompletely captured immigrant status, language, occupation, work conditions, and paid leave.	Research/methods direction: Develop and validate privacy-protective collection of preferred language, occupation, work conditions, paid leave, and treatment disruption; implementation effects remain untested.	Research direction: Evaluate whether standardized collection improves care without increasing immigration-related risk.

Counts reflect assignment of each mapped source to one primary analytic domain; sources could inform additional domains narratively. Direct evidence required an occupationally defined immigrant-worker population. Partial evidence required both an explicitly immigrant-defined population and a substantive work-related construct. Indirect evidence did not meet both conditions. Evidence-supported denotes an intervention or care-delivery practice directly evaluated in the mapped evidence; evidence-informed denotes a reasonable adaptation from related evidence not directly tested in immigrant workers; research or policy direction denotes an action based mainly on evidence gaps or limited direct oncology evidence. Labels apply to individual actions, not entire domains, and do not represent formal certainty grading.

## 3 Results: evidence map

### 3.1 Overview of included evidence

The revised evidence map included 48 sources: the 47 submitted sources plus the July 2026 Department of Homeland Security final rule on public charge (1–47, 51). One source (2.1%) provided direct evidence, 8 (16.7%) provided partial evidence, and 39 (81.3%) provided indirect evidence. The source set comprised 20 empirical studies, 17 evidence syntheses, 9 government, policy, or legal sources, and 2 commentaries or methods papers.

When each source was assigned to one primary domain, structural and policy access accounted for 12 sources, screening and navigation for 12, language and communication for 10, psychosocial care, survivorship, and palliative care for 7, diagnosis and treatment continuity for 3, clinical trial access for 2, and data and measurement for 2. These counts indicated where literature was concentrated, not the certainty or effect size of the evidence.

Only one source directly focused on an occupationally defined immigrant-worker population: a community-based participatory breast-cancer training intervention developed with South Florida Latinx farmworkers (40). Eight sources met the partial-evidence rule because they combined an explicitly immigrant, migrant, undocumented, or foreign-born population with substantive discussion or analysis of work conflicts, employment-linked insurance, worker-defined subgroups, competing work responsibilities, reduced earning capacity, or job loss (1, 2, 23, 25, 29, 31, 32, 44). The remaining 39 sources provided indirect evidence because they did not combine an explicitly immigrant-defined population with a substantive worker or employment construct. This distribution reinforced that most recommendations required cautious application of adjacent evidence or prospective evaluation in worker-defined populations.

### 3.2 Structural and policy access (n=12)

Twelve sources were assigned primarily to structural and policy access: 3 partial and 9 indirect. They consistently described insurance exclusion, underinsurance, fragmented safety-net pathways, cost, transportation difficulty, limited access to primary and specialty care, public-charge concerns, and variable Medicaid or charity-care mechanisms (1–6, 41, 42, 44, 46, 47, 51). The policy evidence was strong for describing eligibility and coverage context but did not establish the effectiveness of worker-specific oncology interventions.

Across these sources, occupational precarity plausibly intensified access barriers through lack of paid leave, unstable schedules, employer-sponsored insurance gaps, and fear of job loss (1, 2). However, the mapped evidence rarely measured whether patients delayed biopsy, missed infusion, interrupted radiation, or discontinued treatment because of wage loss or employment risk. Employer-level interventions and workplace protections remained major evidence gaps.

### 3.3 Language and communication (n=10)

Ten indirect sources were assigned primarily to language and communication. Reviews and empirical studies showed that language barriers affected informed consent, symptom assessment, treatment education, patient experience, and care quality (9–16, 37, 38). Professional interpretation reduced clinically consequential errors compared with *ad hoc* or absent interpretation (12–14), and oncology-specific work supported language-concordant communication and institutional language-access policies (15, 38).

No mapped study directly quantified how language barriers interacted with work schedules, missed wages, treatment adherence, or toxicity reporting among immigrant workers. The evidence nevertheless supported professional interpretation and language-concordant communication as core care-delivery infrastructure, while worker-specific adaptations required evaluation.

### 3.4 Screening and navigation (n=12)

Twelve sources were assigned primarily to screening and navigation: 1 direct, 3 partial, and 8 indirect. They documented screening disparities and barriers among diverse immigrant populations and evaluated culturally tailored education, patient navigation, community health worker outreach, and community-based participatory approaches (17, 23–30, 39, 40, 43). Navigation increased breast, cervical, and colorectal cancer screening among immigrants, and community-engaged interventions improved screening in specific immigrant communities (39, 43).

This domain contained the clearest worker-specific evidence through the Latinx farmworker intervention-development study (40). Even here, evidence was concentrated in outreach and screening rather than diagnostic completion, treatment, or longitudinal cancer outcomes. Worksite-adjacent screening, employer partnerships, and worker-center delivery models remained promising but insufficiently evaluated.

### 3.5 Diagnosis and treatment continuity (n=3)

Three indirect sources were assigned primarily to diagnosis and treatment continuity. Migrants were less likely than non-migrants to receive an early-stage diagnosis overall (7), and nativity and limited English proficiency were associated with concerns about cancer-care quality and communication (8–10). Treatment navigation had supportive evidence in broader oncology populations (18).

The source set did not provide direct estimates of time to diagnosis, time to treatment, radiation interruptions, dose delays, missed infusions, or treatment discontinuation attributable to work constraints among immigrant workers. Bundled visits, predictable calendars, flexible scheduling, and proactive missed-visit outreach were therefore evidence-informed adaptations rather than directly tested worker-specific interventions.

### 3.6 Psychosocial care, survivorship, and palliative care (n=7)

Seven sources were assigned primarily to psychosocial care, survivorship, or palliative care: 2 partial and 5 indirect. The literature described stigma, mistrust, isolation, limited health literacy, psychological distress, end-of-life communication barriers, and inequities in care aligned with patient values (31–36, 45). Psychosocial interventions and interpreter-supported palliative communication had supportive evidence in immigrant or limited-English-proficiency populations (35, 36).

Worker-specific survivorship evidence was especially sparse. Return to work, persistent symptoms, rehabilitation access, job loss, paid leave, retaliation, and workplace accommodation were rarely measured. The evidence therefore supported culturally and linguistically responsive supportive care, while structured return-to-work and workplace interventions remained priorities for prospective study.

### 3.7 Clinical trial access (n=2)

Two indirect sources were assigned primarily to clinical trial access. They documented English-language eligibility requirements and underrepresentation of patients with limited English proficiency in gynecologic oncology trials (19, 20). Reducing avoidable language-based exclusions and providing interpreter-supported consent and translated materials were therefore evidence-informed actions rather than interventions directly tested in immigrant workers. The mapped evidence did not directly measure immigrant-worker enrollment, work-schedule constraints, paid leave, or transportation barriers.

### 3.8 Data and measurement (n=2)

Two indirect sources were assigned primarily to data and measurement. They showed limitations in cancer datasets and registries relevant to race, ethnicity, immigrant status, and equitable interpretation of large datasets (21, 22). Occupation, employment conditions, paid leave, documentation-related vulnerability, and work-related treatment disruption were generally absent. As a result, immigrant workers could not be reliably identified or compared in most existing oncology datasets.

## 4 Public charge policy and cancer care access

The public-charge ground of inadmissibility is a U.S. immigration policy used to assess whether certain noncitizens applying for admission or adjustment of status are likely to become a public charge. It does not apply to every immigrant or every immigration pathway. Historically, determinations focused on cash assistance for income maintenance and long-term institutional care.

In 2019, the federal government expanded the framework to consider additional public benefits, generating widespread concern that use of health or social benefits could affect immigration applications (5, 46). The 2019 rule ceased to operate, and a 2022 final rule restored a narrower framework that excluded many noncash health-related benefits from consideration (6, 46). Studies nevertheless documented persistent avoidance of Medicaid enrollment after the 2019 changes were reversed, consistent with an enduring chilling effect (5).

On July 20, 2026, the Department of Homeland Security published a final rule rescinding the 2022 regulatory framework, effective September 18, 2026 (51). At the time of this revision, the 2022 rule remained operative until the new rule’s effective date; the rescission did not replace it with a new regulatory definition and indicated that further implementation would rely on statute, case law, and agency policy guidance. This transition increased uncertainty and made older static patient-education materials potentially unreliable.

For oncology programs, public-charge relevance extends beyond formal eligibility. Fear or misinformation may discourage eligible patients or mixed-status families from pursuing Medicaid, charity care, financial assistance, or hospital-based services, potentially delaying screening, diagnosis, and treatment. Programs should provide multilingual, date-stamped education developed with legal or policy expertise, update materials when federal rules change, and refer patients for individualized legal advice. Patients should not be required to disclose documentation status to obtain cancer navigation, interpreter services, or social-needs screening.

## 5 Actionable interventions for oncology systems

The actions below were separated by provenance and labeled individually. Evidence-supported denotes an intervention or care-delivery practice directly evaluated in the mapped evidence. Evidence-informed denotes a reasonable adaptation from related evidence that was not directly tested in immigrant workers. Research or policy direction denotes an action based mainly on an evidence gap or limited direct oncology evidence. This taxonomy describes the origin of each action and should not be interpreted as formal certainty grading.

### 5.1 Clinician-level actions

Evidence-supported in adjacent limited-English-proficiency care-delivery studies: professional interpretation reduced clinically consequential errors compared with *ad hoc* or absent interpretation (12–14), and language-concordant oncology communication had supportive evidence (15). Evidence-informed for immigrant workers: routinely integrate these practices into diagnosis disclosure, informed consent, treatment education, toxicity management, survivorship planning, clinical-trial consent, and goals-of-care discussions because they were not directly tested in worker-defined populations. Family members and untrained staff should not substitute for professional interpretation in high-stakes encounters.

As an evidence-informed adaptation, clinicians can ask about preferred language, transportation, insurance, caregiving, work schedule, ability to miss work, and medication access without requiring disclosure of documentation status. Treatment calendars, work letters, activity restrictions, and referrals to navigation, financial counseling, social work, or medical-legal resources can be provided when employment or legal concerns affect care.

### 5.2 Clinic-level actions

Evidence-supported in immigrant screening populations: patient navigation increased breast, cervical, and colorectal screening (43), while broader oncology reviews supported navigation across the care continuum (17, 18). Evidence-informed for immigrant workers: integrate navigation, preferred-language reminders, interpreter-supported teaching, simplified treatment calendars, and proactive outreach after missed visits into worker-facing oncology workflows.

Bundled imaging, laboratory testing, oncology visits, social work, and financial counseling on the same day, along with early-morning, evening, or weekend appointments where feasible, were evidence-informed adaptations to reduce wage and transportation burdens. Their effects on missed visits, treatment completion, and equity should be measured.

### 5.3 Health-system-level actions

Evidence-informed: health systems can create explicit uninsured and underinsured oncology pathways linking abnormal screening to diagnosis, staging, treatment planning, charity-care review, emergency Medicaid assessment where applicable, medication assistance, navigation, and interpreter access. Because these pathways were not directly tested in immigrant workers, they should be implemented with prospective equity metrics rather than assumed to be effective.

Structured data collection can include preferred language, interpreter need, occupation or industry, work-schedule constraints, paid-leave access, transportation, insurance, and social needs with strict privacy protections. Systems can monitor time to diagnosis and treatment, missed visits, treatment interruptions, palliative referral, survivorship planning, and trial enrollment without collecting documentation status unless clinically or legally necessary.

### 5.4 Community and workplace strategies

Evidence-supported in immigrant screening populations: culturally tailored screening, patient navigation, community health worker outreach, and community-based participatory approaches improved screening or screening-related outcomes (28, 39, 43). The farmworker source directly supported participatory development of a community health worker training intervention, but did not evaluate longitudinal screening or cancer outcomes (40). Evidence-informed for immigrant workers: partner with worker centers, unions, consulates, faith organizations, schools, immigrant-serving groups, and legal-aid organizations, provided screening is linked to diagnostic follow-up and oncology navigation.

Worksite-adjacent screening, paid medical leave, job-protected treatment time, anti-retaliation protections, and workplace accommodations were research or policy directions rather than established oncology interventions in the mapped evidence. Pilot programs should measure diagnostic completion, treatment interruption, financial toxicity, job retention, and return-to-work outcomes.

### 5.5 Policy and research-system actions

Research or policy direction: state and federal levers include insurance eligibility, safety-net and charity-care financing, reimbursement for interpretation and navigation, paid medical leave, anti-retaliation enforcement, and language-access standards. Professional societies can develop minimum language-access and work-barrier screening standards, model multilingual materials, and quality measures. Payers can reimburse interpretation and navigation rather than relying on cross-subsidization by individual practices.

For clinical trials and care-delivery research, the National Cancer Institute and the NCI Community Oncology Research Program can support pragmatic studies in community settings, require transparent reporting of preferred language and interpreter need, encourage collection of occupation and work constraints, and test decentralized or flexible trial procedures (52). These mechanisms can connect national research infrastructure with community practices that lack the resources of large cancer centers.

## 6 Discussion

This evidence map showed that immigrant workers were highly relevant to oncology equity but poorly characterized as a distinct population. Only 1 of 48 mapped sources directly focused on an occupationally defined immigrant-worker population, 8 met the two-condition partial-evidence rule, and 39 were indirect. Most evidence therefore came from adjacent immigrant, undocumented, foreign-born, uninsured, policy, or limited-English-proficiency populations, making explicit evidence provenance essential.

The clearest intervention evidence—derived mainly from adjacent limited-English-proficiency or broader immigrant populations—supported professional interpretation, language-concordant communication, patient navigation, community health worker outreach, culturally tailored screening, and community-based participatory approaches (12–18, 28, 39, 40, 43). Applying these approaches to immigrant-worker oncology care was evidence-informed except where an immigrant intervention was directly evaluated; the sole worker-specific source evaluated intervention development rather than longitudinal cancer outcomes (40). Flexible scheduling, bundled visits, employer engagement, medical-legal partnerships, and structured return-to-work support remained research or policy directions requiring prospective evaluation.

Under-measurement likely reflected several mechanisms. Immigrant worker was inconsistently defined; oncology datasets rarely collected occupation, employment conditions, or paid leave; documentation-related questions raised privacy and mistrust concerns; and seasonal, informal, or changing employment complicated longitudinal follow-up (21, 22). Limited dedicated funding and small subgroup sizes further reduced analytic visibility. A minimum worker-relevant dataset could prioritize preferred language, interpreter need, occupation or industry, work schedule, paid leave, job protection, transportation, and treatment disruption while avoiding unnecessary collection of documentation status.

Implementation capacity varied substantially. Smaller community practices may be unable to build dedicated navigation, legal, financial, and extended-hours programs. A tiered approach is therefore more realistic: low-resource practices can prioritize reliable remote or in-person interpretation, preferred-language documentation, brief work-barrier screening, coordinated scheduling, and referral links; larger systems can add dedicated navigation, uninsured pathways, extended hours, medical-legal partnerships, and equity dashboards. Regional hubs, payers, professional societies, and NCORP networks can provide shared infrastructure, standardized materials, and implementation support.

Responsibility is distributed across a complex system. Clinicians and clinic leaders control communication and scheduling workflows; health-system executives allocate navigation, interpreter, and financial-assistance infrastructure; payers and state programs determine reimbursement and coverage pathways; employers, labor agencies, and legislatures shape paid leave and job protection; professional societies establish standards; and NCI and NCORP can fund and normalize worker-relevant data collection and pragmatic trials. Naming these actors converts broad recommendations into identifiable levers for action.

The July 2026 public-charge rescission illustrated why oncology guidance must be dynamic. Even when legal rules change, fear and misinformation may persist, and rapidly changing federal guidance can make static counseling inaccurate. Oncology systems should therefore combine general, date-stamped education with referral to qualified legal resources rather than provide individualized immigration advice.

## 7 Limitations

First, this study used a modified, practice-oriented evidence map rather than a completed PRISMA-ScR scoping review. The search was targeted rather than exhaustive, screening and charting were performed by one author without independent duplication, no protocol was registered, and no formal source-level risk-of-bias or certainty assessment was applied. The source and domain counts therefore describe the mapped literature and should not be interpreted as comprehensive prevalence estimates or evidence grades.

Second, only one source directly focused on an occupationally defined immigrant-worker population, and only eight met the two-condition partial-evidence rule. Most conclusions relied on indirect evidence from broader immigrant, foreign-born, undocumented, uninsured, policy, migrant, or limited-English-proficiency populations, limiting causal inference and worker-specific generalizability.

Third, immigrant workers are heterogeneous by country of origin, language, race and ethnicity, documentation status, occupation, income, insurance, duration of residence, and policy context. Findings from one group or jurisdiction may not transfer to another.

Fourth, included sources varied substantially in design, setting, cancer type, outcome definition, and policy purpose, precluding quantitative pooling. Intervention evidence was concentrated in language access, navigation, screening, and community outreach, whereas flexible oncology scheduling, paid leave, workplace protections, medical-legal partnerships, and return-to-work programs remained understudied.

Finally, the evidence base itself incompletely captured occupation, employment conditions, paid leave, documentation-related vulnerability, and work-related treatment disruption. This measurement gap likely led to under-identification of immigrant workers and limited outcome comparisons.

## 8 Conclusion

Immigrant workers are not simply a subset of immigrant patients. Their cancer-care experiences are shaped by the intersection of employment precarity, lack of paid leave, income loss, legal vulnerability, language barriers, insurance exclusion, and fragmented access. Yet only one mapped source directly focused on an occupationally defined immigrant-worker population.

This evidence map identifies both a research gap and an implementation opportunity. Oncology systems can implement supported language-access, navigation, and community-engagement strategies now; adapt scheduling, coordination, financial, and work-barrier workflows with prospective evaluation; and advocate for coverage, reimbursement, paid-leave, and workplace protections. Future research should directly measure occupation, employment conditions, language, insurance, paid leave, treatment disruption, job retention, and return-to-work outcomes.
